# Mesmerism

**Published:** 1845-03

**Authors:** 


*Mesmerism.*—After the unequivocal and almost unprecedented exposure to which the fraud of mesmerism was subjected, under our own immediate guidance and inspection, we did not consider that it would again be necessary to notice such a piece of arrant trickery and scandal in our columns; but a proper feeling of gallantry demands that we should not pass unnoticed the literary production with which that well-known lady, Miss Martineau, has favored the profession and the public, in the number of the *Athenæum*, journal of literature, for November 23. Miss Martineau, it appears, has long been an implicit believer in the powers of "mesmerism," and yet omitted to resort to that art for aid, although laboring, as she believed, under an incurable disease, during a confinement to her room of four and a half years' duration—a circumstance which seems to us to be entirely inexplicable, and renders the statement of her suffering for so long a period, and her firm belief in the efficacy of the remedial agency of mesmerism, utterly inconsistent and irreconcilable. If Miss Martineau had been considered to be a quack in politics and literature, her present performance might have been regarded as unworthy of attention. It might have been considered, that in producing the essay now before us, she had not manifested any unusual extravagance of thought, pretension, or feeling. But, inasmuch as she has attracted much notice, and the opinions of the world are strikingly divided as to the character of her mental and literary labors, we apprehend that her recent performance in mesmerism will be attended with a different result, and that only one opinion will or can prevail concerning its true nature and objects. To the sober judgment, then, of our medical readers, we commit the following extracts from an essay on mesmerism, by an aged maiden lady:

"One very warm morning in August, when everybody else was oppressed with heat, I was shivering a little under the mesmeric influence of my maid—the influence, in those days, causing the sensation of cold currents running through me, from head to foot. 'This cold will not do for you, ma'am,' said M. 'O!' said I, 'it is fresh, and I do not mind it;' and immediately my mind went off to something else. In a few minutes I was surprised by a feeling of warm water trickling through the channels of the late cold. In reply to my observation, that I was warm now, M. said, 'Yes, ma'am, that is what I am doing.' By inquiry and observation, it became clear to me, that her influence was, generally speaking, composing, just in proportion to her power of willing that it should be so."—*Athenæum*, No. 891, pages 1071–72.

"As the muscular power oozes away under the mesmeric influence, a strange inexplicable feeling ensues of the frame becoming transparent and ductile. My head has often appeared to be drawn out, to change its form, according to the traction of my mesmerist, and an indescribable and exceedingly agreeable sensation of transparency and lightness, through a part or the whole of the frame, has followed. Then begins the moaning, of which so much has been made, as an indication of pain. I have often moaned, and much oftener have been disposed to do so, when the sensations have been most tranquil and agreeable. At such times, my mesmerist has struggled not to disturb me by a laugh, when I have murmured, with a serious tone, 'Here are my hands, but they have no arms to them;' 'O dear! what shall I do? here is none of me left!' the intellect and moral powers being all the while at their strongest. Between this condition and the mesmeric sleep there is a state, transient and rare, of which I have had experience, but of which I intend to give no account. A somnambule calls it a glimmering of the lights of somnambulism and clairvoyance. To me there appears nothing like glimmering in it. The ideas that I have snatched from it, and now retain, are, of all ideas which ever visited me, the most lucid and impressive. *It may be well that they are incommunicable*—partly from their *nature* and *relations*, and partly from their *unfitness for translation into mere words.* I will only say that the condition is one of no 'nervous excitement,' as far as experience and outward indications can be taken as a test. Such a state of repose, of calm translucent intellectuality, I had never conceived of; and no reaction followed, no excitement but that which is natural to every one who finds himself (query, *herself*) in possession of *a great new idea.*"—*Idem*, page 1072.

[The following letter from Dr. Robert Hull, of Norwich, where Miss M. resides, has more recently appeared.]

"This admired writer has, however, thought right to announce her case publicly as one of successful mesmerism—and the interests of truth and society compel the antagonists of this medical heresy to analyze, so far as possible, the history, and falsify the conclusion, that, because the patient is well, the mesmeric aura has effected her cure. Now, although the laudable delicacy of this extraordinary lady hath suppressed the details of her malady, yet I have a right to assume that the circulated whispers were well founded, and that the malady was abdominal tumor. Here (in Norwich) this celebrated author is too well known that her age can be any secret; and her amiable and simple character would render her careless to conceal. And she will not be surprised, therefore, nor angry, if she is told, that she has been laboring under the climacteric disorder of her sex; that this often produces a physcony of the abdomen, with oppression and universal languor; that in such circumstances the single woman is terrified with ideas of cancer, dropsy, and organic diseases; the married lady fancies she is about to multiply the species, and her fond husband provides a doctor and the nurse. A case of this kind is reported in the person of a Mrs. Trunnion, by Dr. Smollett; and instances of the first-named deception in spinsters are daily occurring. But nature goes through her proceedings; the abdominal tumefactions subside; and when the climacteric period has passed, women often enjoy better health and longer life than the other sex. In this particular case of our popular townswoman let not the mesmerizer triumph! The success was due to the natural process, aided by the vigor obtained from faith and hope. Hence energy, exercise, air, *omission of opiates*—and it seems to me that this delightful result would have been earlier effected—I mean the natural cure—had not the patient become, from her own confession, a complete opium-eater. She had poiscned herself for years with this exterminating drug. To conclude: my firm persuasion is, that this vaunted case is one of thousands, in which the mind has relieved the body from *functional*, not organic disorders; while ladies of a particular age will do well *not* to applaud Mesmer for the cure of their peculiar symptoms, which time and the physician will generally cure, unless baffled by pernicious treatment; and that young ladies should be especially careful to eschew this revived foolery, which in many instances hath created, instead of relieved, tumors of the abdomen."—*London Lancet.*

